# Inter- and intradialytic fluid volume changes and vascular stiffness parameters in patients on hemodialysis

**DOI:** 10.1371/journal.pone.0262519

**Published:** 2022-02-03

**Authors:** Aya Lafta, Judy Ukrainetz, Sara Davison, Stephanie Thompson, Aminu Bello, Branko Braam

**Affiliations:** 1 Division of Nephrology and Immunology, Department of Medicine, University of Alberta, Edmonton, Alberta, Canada; 2 Alberta Health Services, Alberta Kidney Care-North, University of Alberta Hospital, Edmonton, Alberta, Canada; 3 Department of Physiology, University of Alberta, Edmonton, Alberta, Canada; Universidade Estadual Paulista Julio de Mesquita Filho, BRAZIL

## Abstract

**Background:**

Whether fluid overload is associated with vascular stiffness parameters in hemodialysis (HD) patients has not been fully elucidated. We hypothesized that interdialytic fluid accumulation increases vascular stiffness parameters, which improves with intradialytic ultrafiltration.

**Methods:**

Fluid overload and vascular stiffness parameters were assessed in 39 HD patients (20 with and 19 without fluid overload) and compared to 26 healthy controls. Fluid status was assessed 15 minutes before the mid-week HD session by bio-impedance spectroscopy. Following this, ambulatory pulse wave velocity (PWV) and augmentation index (AIx) were measured for 24 hours before another mid-week HD session and then for 5 hours starting 30 minutes before and ending 30 minutes after the session.

**Results:**

HD patients had significant fluid overload compared to healthy controls (2.0±2.4 vs. -0.2±0.6 L; P<0.001) and baseline PWV was higher (10.3±1.7 vs. 8.8±1.4 m/s; P<0.001). There was no significant difference between PWV and AIx in fluid overloaded and non-fluid overloaded HD patients prior to, or during the HD session. AIx of non-fluid overloaded HD patients improved after the HD session (P = 0.04). Average 24-hour AIx was higher in fluid overloaded HD patients (P<0.001).

**Conclusions:**

Inter- and intradialytic changes in fluid volume were only weakly related to vascular stiffness parameters in HD patients. Although there was a modest reduction in AIx in non-fluid overloaded HD patients after the dialysis session, fluid removal did not improve vascular stiffness parameters during the HD session. We speculate that the effect of fluid overload correction on vascular stiffness parameters requires long-term adjustments in the vasculature.

## Introduction

Patients with end-stage kidney disease treated with hemodialysis (HD) have a high prevalence of fluid overload [1, 2] and have increased vascular stiffness parameters [3, 4]. Previous reports support that fluid overload is strongly associated with poor overall survival rate in HD patients [5, 6]. Pulse wave velocity (PWV) and augmentation index (AIx) are measures of vascular stiffness that are independently associated with a higher risk of cardiovascular events and all-cause mortality [7–9]. The relationship between fluid overload and vascular stiffness parameters in HD patients is not fully elucidated.

Vascular stiffness in HD patients is increased by structural and functional changes. Structural changes, such as atherosclerosis and vascular calcification, presenting as media sclerosis, increase PWV and AIx and increase vascular stiffness [10, 11]. Functionally, increased activity of the renin-angiotensin system, decreased nitric oxide levels, increased oxidative stress, and inflammation, which have all been described in HD patients, can contribute to increased vascular stiffness [12]. It has also been suggested that fluid overload may increase vascular stiffness by increasing arterial wall tension [11]. Furthermore, the cyclic changes in fluid status due to the intermittent nature of HD might decrease arterial compliance and increase vascular stiffness in patients [13].

Altogether, whether fluid overload affects vascular stiffness in HD patients remains unclear. We hypothesized that interdialytic fluid accumulation increases vascular stiffness parameters, which improves with intradialytic ultrafiltration. We aimed to confirm that vascular stiffness parameters are higher in HD patients compared to healthy controls and to investigate the effect of inter- and intradialytic fluid changes on PWV and AIx measurements in fluid overloaded and non-fluid overloaded HD patients.

## Methods

### Study participants

We recruited 39 prevalent HD outpatients from the University of Alberta Hospital, Edmonton Alberta, Canada. Inclusion criteria were 18 years of age or above and dialysis-dependency for more than 6 weeks, with a three times per week frequency and a HD duration of 3.5 to 4 hours per session. Exclusion criteria were acute illnesses that included cardiovascular events and infection, surgery within 6 weeks of the study, pregnancy, nocturnal dialysis, kidney transplantation during the study, implanted pacemakers, defibrillators, pins, metallic stent, artificial joints, and skin lesions at the site where bioimpedance electrodes should be positioned. As far as applicable, the same criteria were used for the healthy subjects. A health questionnaire was obtained from the healthy subjects. Written informed consent was obtained from all participants. The study was approved by the Human Research Ethics Board at the University of Alberta Hospital.

### Assessment of fluid overload

Extracellular fluid volume, intracellular fluid volume and total body fluid volume of all study participants were assessed using a validated, portable bio-impedance spectroscopy device (Body Composition Monitor, BCM^®^ Fresenius Medical Care, Bad Hamburg, Germany) [14, 15]. The reason behind using the bio-impedance is that multifactorial in etiology, and clinical assessment being unreliable to assess fluid volume status [16]. For each assessment, we used the average of three consecutive measurements taken in a quiet room in the dialysis unit with the participants in a supine position. In HD patients with an arteriovenous fistula, the side contralateral to the fistula was used. The measurements were performed 15 minutes before the start of the mid-week HD session. Fluid overload was defined as an excess of fluid volume of ≥1.1 L above normal estimated ECFV, as calculated by the bio-impedance equipment based on a mathematical model [15, 17, 18]. The HD patients were divided into fluid overloaded HD (fluid overload ≥1.1 L of normal ECFV) and non-fluid overloaded HD groups (fluid overload <1.1 L of normal estimated ECFV) [19]. The 1.1 L cut-off point was based on the calculated 10^th^ and 90^th^ percentiles of the healthy distribution to define the normovolemia range which yielded to -1.1L and +1.1L, respectively. Also, fluid overload normally varies from -1.1 L below normal ECFV after HD run to + 1.1 L above normal ECFV before the next HD run with a 75 mmol/d sodium intake [14].

### Technical aspects and timing of vascular stiffness parameters and blood pressure measurements

We used an oscillometric device to measure aortic PWV, brachial AIx, peripheral systolic and diastolic blood pressure, mean arterial pressure, and pulse pressure using an upper arm cuff using the non-fistula arm for HD patients (Arteriograph24^TM^, TensioMed, Budapest, Hungary) [20]. This device measures three parameters: systolic wave, reflected wave and diastolic wave. PWV, AIx, and blood pressure were measured before the mid-week HD session for 24 hours, which was followed by a 5-hour measurement starting 30 minutes before and ending 30 minutes after the dialysis session. In healthy controls, a 5-hour measurement of PWV, AIx and blood pressure was performed as a time control.

### Clinical data

Dialysis vintage, vascular access, ultrafiltration, and current target weight were obtained from the patients’ record. Cardiovascular events like coronary artery diseases and other illnesses were obtained from patient’s medical history reported in the electronic medical record. Interdialytic weight gain was calculated by subtracting the post-dialysis weight of previous HD session from the pre-dialysis weight of the HD session. Patients were considered diabetic if it was documented in the chart or they were prescribed anti-diabetic medications. Antihypertensive medications were obtained from the electronic medical record.

### Statistical analysis

Continuous data are expressed as mean ± standard deviation if normally distributed or as median 25–75 percentile if not normally distributed. Categorical variables are expressed as percentage of total. Shapiro-Wilk test was used to test normal distribution of variables. Chi-square test was used to compare frequencies. Mann-Whitney U test was performed to compare nonparametric parameters between HD group versus healthy controls and between fluid overloaded HD versus non-fluid overloaded HD groups. Two-way repeated measurement ANOVA was used to analyze the variance of vascular stiffness parameters during the HD session, the changes of variables in pre-and post-HD session, and the 24 hours measurements. Tukey multiple comparison test was applied as post-hoc test. The missing data and outliers were imputed by performing a regression analysis using the entire data set of each parameter per subject. Univariate and multivariate linear regression analysis were performed to determine the factors predicting pre-HD PWV. Correlation between non-parametric data were analyzed with Spearman’s test and Pearson’s test was used for parametric data. Graphpad Prism version 8 (Graphpad Software, San Diego, CA, USA) and SPSS version 25 (SPSS Inc., Chicago, IL, USA) were used for data analysis. P<0.05 was considered statistically significant.

## Results

### Baseline characteristics of the study group

HD patients were significantly older than healthy controls and were fluid overloaded. Fluid overloaded HD had higher dialysis vintage than non-fluid overloaded HD patients. Fifty-nine percent of HD patients were on anti-hypertensive medications; beta-blockers were prescribed most frequently. Anti-hypertensive medications were prescribed more commonly in fluid overloaded HD compared to non-fluid overloaded HD patients. Both fluid overloaded HD and non-fluid overloaded HD patients had similar interdialytic weight gain, prevalence of cardiovascular disease (coronary artery disease, myocardial infarction, and heart failure) and hypertension, and causes of end stage renal disease. Demographic and clinical characteristics, the baseline measurements before the 5 hours measurements of all study participants as well as the mean 24h measurements of HD patients are shown in **Table 1**.

**Table 1:** Demographic and clinical characteristics in healthy controls and HD patients.

Parameters	Healthy controls (n = 26)	HD group (n = 39)	P value	FO HD (n = 20)	non-FO HD (n = 19)	P value
Sex M N, %	10 (38.4%)	24 (61.5%)	0.06	14 (70%)	10 (52.6%)	0.26
Age, year	49 (29–56)	60 (49–66)	0.006	62 (51–68)	57 (21–67)	0.05
Body mass index, kg/m^2^	23.9 ± 3.5	27.0 ± 5.6	0.008	26.2 ± 4.9	27.9 ± 6.2	0.33
Weight, kg	68.2 ± 10.9	78.9 ± 17.4	0.003	77.9 ± 16.9	79.9 ± 18.4	0.72
Height, cm	168.4 ± 6.8	170.0 ± 10.7	0.50	171.2 ± 10.5	168.8 ± 11.2	0.44
Post-HD weight, kg	-	77.0 ± 17.7	-	75.8 ± 16.9	78.2 ± 18.0	0.66
Underlying cause of ESRD N, %						
Diabetes						
Hypertension						
Polycystic kidney disease						
Glomerulonephritis						
Unknown						
Other						
FO, L	-0.2 ± 0.6	2.0 ± 2.4	<0.0001	3.7 ± 2.3	0.1 ± 0.5	< 0.0001
FO post-HD, L	-	0.0 ± 2.2	-	1.6 ± 1.9	-1.6 ± 1.0	<0.0001
ECFV, L	15.10 ± 2.1	17.88 ± 4.2	0.001	19.17± 4.2	16.54 ± 3.9	0.05
ICFV, L	19.08 ± 19.0	18.30 ± 18.3	0.482	17.55 ± 4.0	19.09 ± 4.7	0.28
TBFV, L	34.18 ± 5.9	36.10 ± 8.1	0.287	36.72 ± 7.9	35.63 ± 8.5	0.69
FO/ECFV, %	-0.7 (-4.5–3.1)	7.1 (1.6–19.5)	<0.0001	18.8 (10.0–24.9)	1.6 (-2.0–3.7)	<0.0001
IDWG, kg	-	1.3 (0.0–3.4)	-	1.4 (0.87–2.0)	1.2 (0.6–2.1)	0.66
Dialysis vintage, years	-	2.0 (0.1–12.0)	-	3.0 (2.0–4.0)	1.0 (0.3–3.0)	0.02
Net ultrafiltration, L	-	1.9 ± 1.1	-	2.2 ± 1.1	1.7 ± 1.0	0.19
Anuric N, %	-	13 (33%)	-	4 (20%)	9 (47%)	0.07
Diabetes N, %	-	10 (2.5%)	-	6 (30%)	4 (21%)	0.52
Cardiovascular diseases N, %	-	21 (53%)	-	10 (50%)	11(57%)	0.61
Anti-hypertensive medications N, %	-	23 (59%)	-	15 (75%)	8 (42%)	0.03
Beta blockers N, %	-	18 (46%)	-	14 (70%)	4 (21%)	0.25
Calcium channel blockers N, %	-	11 (28%)	-	9 (45%)	3 (15.7%	0.16
Diuretics N, %	-	7 (18%)	-	5 (25%)	2 (10.5%)	0.21
Angiotensin receptor blockers N, %	-	5 (12.8%)	-	4 (20%)	1 (5.2%)	0.40
Baseline systolic blood pressure, mmHg	117.0 ± 11.9	138.2 ± 22.2	< 0.0001	144.4 ± 21.5	131.7 ± 21.5	0.07
Baseline diastolic blood pressure, mmHg	69.3 ± 11.3	76.1 ± 15.5	0.04	76.8 ± 11.0	75.4 ± 19.5	0.78
Baseline mean arterial pressure, mmHg	85.2 ± 10.8	95.7 ± 15.4	0.002	99.4 ± 13.0	91.7 ± 17.2	0.12
Baseline heart rate, bpm	69.6 ± 12.1	79.7 ± 12.2	0.001	78.7 ± 12.2	80.8 ± 12.6	0.70
Baseline pulse pressure, mmHg	49.9 ± 9.9	61 ± 15.8	<0.001	60.0 ± 11.5	50.9 ± 15.3	0.04
Baseline PWV, m/s	8.8 ± 1.4	10.3 ± 1.7	<0.001	10.3 ± 1.4	10.2 ± 1.9	0.83
Baseline AIx, %	-22.3 ± 27.1	-10.3 ± 36.6	0.13	-14.8 ± 30.7	-5.5 ± 42.2	0.44
24hr-mean Systolic blood pressure, mmHg	-	131.6 ± 19.5	-	138.2 ± 18.0	123.5 ± 17.2	0.01
24hr-mean Diastolic blood pressure, mmHg	-	70.8 ± 11.5	-	72.1 ± 8.8	68.8 ± 13.4	0.37
24hr-mean arterial pressure, mmHg	-	90.4 ± 12.9	-	99.4 ± 13.0	91.7 ± 17.2	0.01
24hr-mean heart rate, bpm		78.5 ± 8.6		77.8 ± 10.4	79.2 ± 6.3	0.66
24hr-mean Pulse pressure, mmHg		60.4 ± 13.4	-	65.8 ± 14.4	54.7 ± 9.8	0.008
24hr-mean PWV, m/s	-	9.9 ± 1.1	-	9.9 ± 1.1	9.8 ± 1.3	0.80
24hr-mean AIx, %	-	-12.4 ± 22.3	-	-1.4 ± 21.9	-25.6 ± 18.1	<0.001

P value < 0.05 is considered significant; ESRD, end stage renal disease; FO, fluid overload; ECFV, extracellular fluid volume; ICFV, intracellular fluid volume; TBFV, total body fluid volume; PWV, pulse wave velocity; AIx, augmentation index; IDWG, intradialytic weight gain. FO post-HD = FO pre-HD- net ultrafiltration.

### Blood pressure and vascular stiffness parameters in study groups

**Fig 1:**
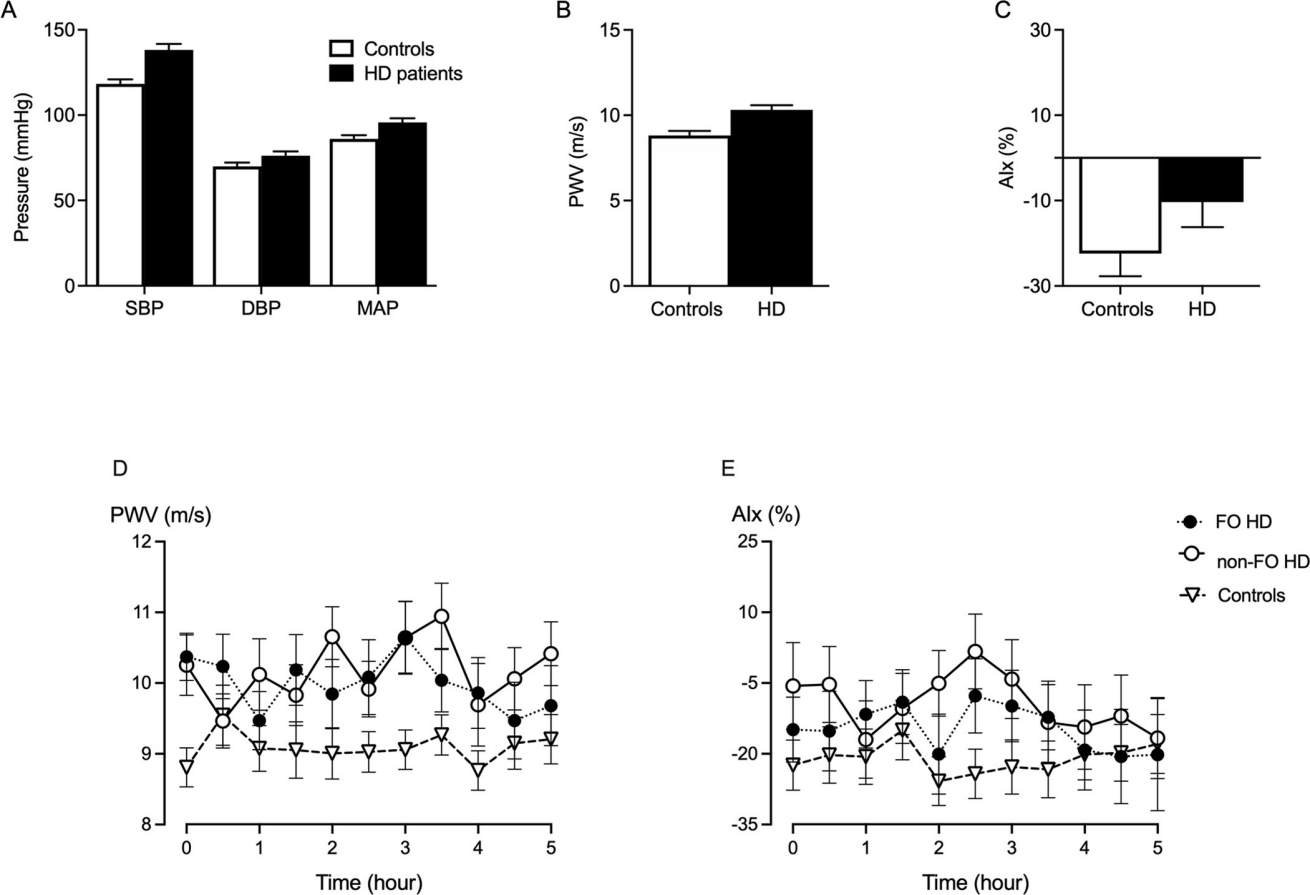
Results of blood pressure and vascular stiffness parameters at the baseline and over 5-hours in HD patients and healthy controls. Significant differences in baseline systolic blood pressure (SBP), mean arterial pressure (MAP), and diastolic blood pressure (DBP), were observed in HD patients (black bar) compared to healthy controls (white bar) (A). Baseline pulse wave velocity (PWV) was significantly higher in HD patients than in healthy controls (B). Augmentation index (AIx) was not different between the two groups (C). No significant changes in PWV (D) and AIx (E) were observed during the 5-hours measurements in fluid overloaded HD (closed symbol; dash dotted-line) and non-fluid overloaded HD patients (opened symbol; solid line). That was matching with the results of healthy controls (inverted triangle; dash-solid lines).

HD patients had significantly higher systolic blood pressure, mean arterial pressure, pulse pressure, and PWV values compared to healthy controls as assessed from the measurements immediately prior to the HD session for HD patients or at the start of the 5 hours measurements in the healthy controls. Pulse pressure was higher in fluid overloaded HD than non-fluid overloaded HD patients, however, systolic blood pressure, diastolic blood pressure, and mean arterial pressure were not different. The PWV and AIx were also not different between fluid overloaded HD and non-fluid overloaded HD patients as well (**Table 1, Fig 1A–1C**). After the HD session, fluid overload remained significantly higher in fluid overloaded HD compared to non-fluid overloaded HD patients. Over the 5 hours of measurements, no significant changes in PWV and AIx were found in both HD groups and that was similar to healthy controls (**Fig 1D and 1E**). In healthy individuals over 49 years of age, PWV and AIx were significantly higher than in younger individuals **S1 Table**. Multivariate analysis showed that advanced age was an independent predictor of high PWV in healthy individuals **S2 Table**. After univariate linear regression analysis of baseline PWV predictors, extracellular fluid volume and total body fluid volume were included in the multivariate analysis. In the multivariate analysis, neither extracellular fluid volume nor total body fluid volume were independent predictors for baseline PWV in HD patients **S3 Table**.

### Was ultrafiltration volume related to improvements in vascular stiffness parameters?

Ultrafiltration volume in fluid overloaded and non-fluid overloaded HD patients was similar. There was a significant reduction in AIx in non-fluid overloaded HD patients compared to the AIx values at the start of the HD session. PWV values were unchanged after the HD session in both fluid overloaded and non-fluid overloaded HD patients **S4 Table**.

To test whether fluid removal by ultrafiltration volume improves PWV and AIx, we performed a simple linear regression analysis. There was a positive relationship between ultrafiltration volume and post-HD pulse pressure in fluid overloaded HD patients and between ultrafiltration volume and the change in PWV in non-fluid overloaded patients. Ultrafiltration volume and post-HD AIx showed a tendency for a positive correlation in fluid overloaded HD patients. However, regression analysis did not reveal a significant relationship between ultrafiltration volume and post-HD systolic blood pressure, diastolic blood pressure, mean arterial pressure, and change in AIx in fluid overloaded and non-fluid overloaded HD patients **S5 Table**.

### Does interdialytic fluid accumulation affect blood pressure and vascular stiffness parameters?

**Fig 2:**
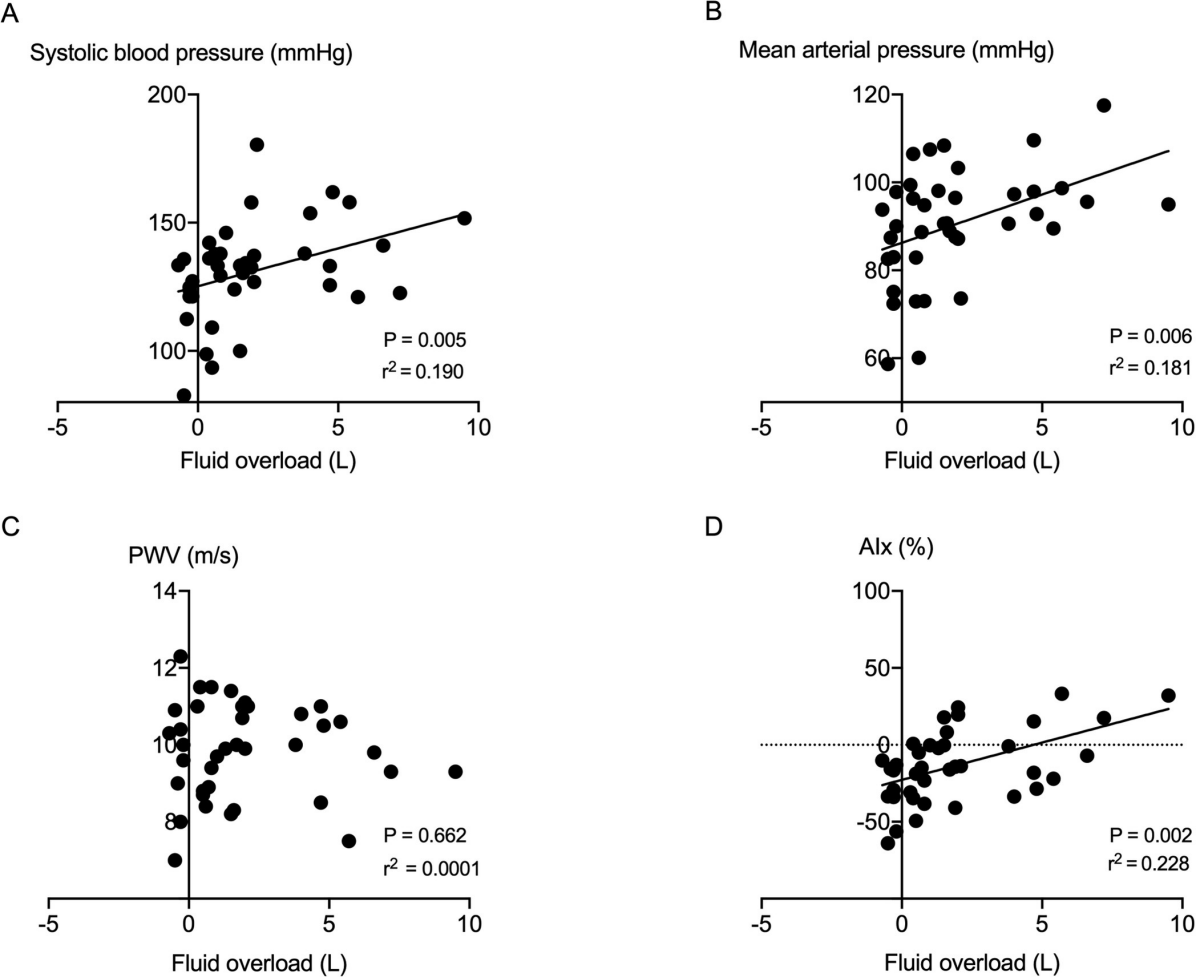
Correlation analysis between fluid overload and interdialytic ambulatory measurements of blood pressure and vascular stiffness parameters in HD patients. Fluid overload was positively correlated with the 24-hour mean systolic blood pressure (SBP; A), mean arterial pressure (MAP; B), and augmentation index (AIx; D), but it was not correlated with the 24-hour mean pulse wave velocity (PWV; C).

Average 24-hour systolic blood pressure, mean arterial pressure and pulse pressure were significantly higher in fluid overloaded HD patients than non-fluid overloaded HD patients. AIx was higher in fluid overloaded versus non-fluid overloaded HD patients, yet average PWV was not different (**Table 1, Fig 2A–2C**).

**Fig 3:**
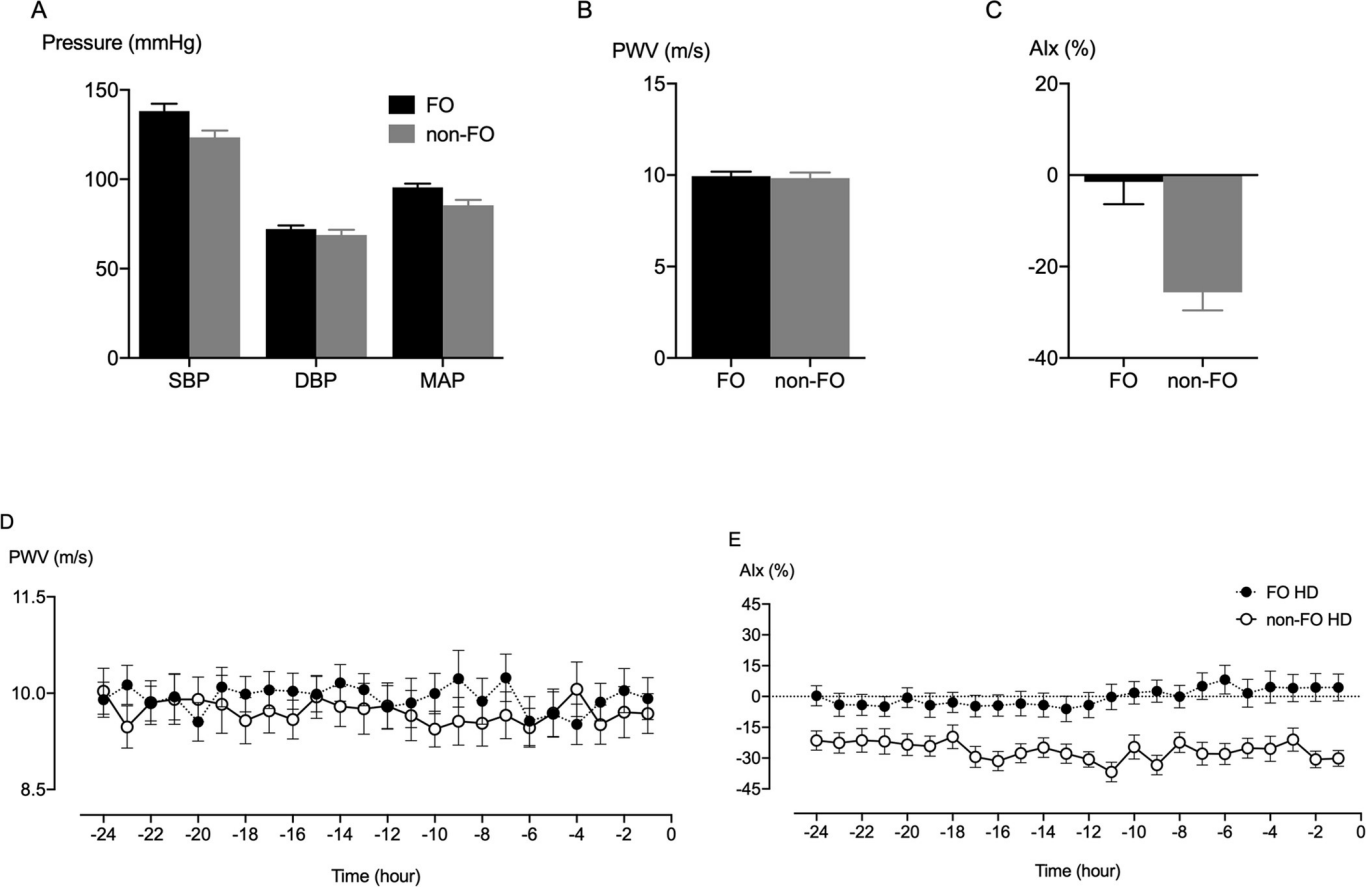
Results of the ambulatory interdialytic assessments of blood pressure and vascular stiffness metrics in fluid overloaded and non-fluid overloaded HD patients. The 24-hour mean systolic blood pressure (SBP) and mean arterial pressure (MAP), but not diastolic blood pressure (DBP), were higher in fluid overloaded HD (black bar) versus non-fluid overloaded HD patients (grey bar) (A). No significant difference in the 24-hour mean pulse wave velocity (PWV) was shown between fluid overloaded HD and non-fluid overloaded HD patients (B). The 24-hour mean augmentation index (AIx) was higher in fluid overloaded HD than non-fluid overloaded HD patients (C). There were no clear changes in the 24-hour PWV (D) and AIx (E) in the two HD groups.

Univariate linear regression analysis regarding average interdialytic AIx revealed mean arterial pressure, fluid overload, and fluid overload/extracellular fluid volume, extracellular fluid volume /total body fluid volume, and extracellular fluid volume /intracellular fluid volume ratios as potential predictors. In the multivariate analysis, mean arterial pressure was the only significant predictor for average interdialytic AIx **Table 2**. A significant relationship was observed between fluid overload and inter-dialytic systolic blood pressure, mean arterial pressure, and AIx in HD patients. There was no association with interdialytic PWV (**Fig 3A–3D**).

**Table 2:** Predictors of 24-mean AIx in HD patients using univariate and multivariate linear regression analysis.

Parameters	Univariate analysis			
	B	T	CI (95%)	P value
Age, year	0.112	0.066	-0.453 to 0.676	0.69
Pre-HD weight, kg	-0.273	-1.239	-0.720 to 0.173	0.22
IDWG (kg)	3.025	0.667	0.509 to 12.215	0.50
FO, L	5.217	3.907	2.512 to 7.923	<0.001
ECFV, L	0.868	0.954	-0.976 to 0.45	0.34
ICFV, L	-0.626	-0.711	-2.411 to 1.158	0.48
TBFV, L	0.40	0.084	0.934 to 1.017	0.93
FO / ECFV, %	1.194	4.331	0.636 to 1.753	<0.001
ECFV/TBFV, %	2.288	2.620	0.518 to 4.057	0.01
ECFV/ICFV, %	0.594	2.685	0.146 to 1.042	0.01
Systolic blood pressure, mmHg	0.031	0.150	-0.387 to 0.449	0.88
Diastolic blood pressure. mmHg	0.288	0.860	-0.391 to 0.967	0.39
Mean arterial pressure, mmHg	0.966	3.613	0.424 to 1.508	0.001
Pulse pressure, mmHg	-0.115	-0.389	-0.71 to 0.483	0.70
Dialysis vintage, years	1.748	1.227	-1.13 to 4.63	0.22
	Multivariate analysis			
FO, L	-0.444	-0.099	-9.61 to 8.69	0.92
FO / ECFV, %	1.154	1.114	-0.0094 to 0.032	0.27
ECFV/TBFV, %	-3.943	-0.375	-25.3 to 17.4	0.71
ECFV/ICFV, %	0.850	0.310	-4.736 to 6.437	0.75
Mean arterial pressure, mmHg	0.656	2.380	0.095 to 1.217	0.02

P value < 0.05 is considered significant; IDWG, intradialytic weight gain; FO, fluid overload; ECFV, extracellular fluid volume; ICFV, intracellular fluid volume; TBFV, total body fluid volume.

## Discussion

We investigated inter- and intradialytic fluid changes and vascular stiffness parameters in HD patients. First, we confirmed that HD patients have higher vascular stiffness parameters than healthy controls, which is consistent with other publications [11, 21, 22]. Second, we found that the effect of ultrafiltration volume on AIx, was more pronounced in non-fluid overloaded versus fluid overloaded HD group. Third, our results showed that the 24-hour interdialytic PWV and AIx remained unchanged in the two HD groups, although interdialytic AIx was higher in fluid overloaded HD compared to the non-fluid overloaded HD patients.

Very few studies have evaluated vascular stiffness parameters in HD patients with varying levels of fluid overload [23, 24]. One study found that the pre-HD PWV was not different among hypervolemic, normovolemic and hypovolemic HD patients [23]. In contrast, another study found that the pre-HD PWV was higher in fluid overloaded versus non-fluid overloaded HD patients [23, 24]. Different definitions of fluid overload as well as different characteristics of the dialysis patients, such as dialysis vintage, medications and co-morbidities limit the comparison between our current results and previous reports. However, our findings do not indicate that fluid overload is a strong predictor of vascular stiffness parameters in HD patients.

We then investigated whether *intradialytic* fluid removal would acutely improve vascular stiffness parameters in HD patients. We found that PWV and AIx did not change during the HD run when all patients were considered. A previous study observed a significant decline in intradialytic PWV only at 135 and 210 minutes into the HD run, whereas AIx remained unchanged [25]. The latter study did not provide information about the fluid volume status of the HD patients, and the two time points at which PWV displayed a significant change were associated with a blood pressure reduction. Furthermore, it has been reported that HD treatment may reduce AIx following the HD session [11, 24, 26]. In our study, we only found a modest significant reduction in AIx in non-fluid overloaded HD after the HD run. Our results were are in line with a study that found a significant reduction in AIx values in non-fluid overloaded compared to fluid overloaded HD patient after the HD run [24]. Three more studies showed a significant reduction in AIx after the mid-week HD session with unchanged PWV in HD patients regardless of their fluid status [11, 26, 27]. In our study, we found that fluid volume overload remained more than 1.1 L in fluid overloaded HD patients and lower than 1.1 L in non-fluid overloaded HD patients after the HD session. As discussed, fluid overload increases pulse pressure in HD patients [26] and pulse pressure is a component of AIx. This could explain why AIx was reduced after HD run in non-fluid overloaded HD and remained unchanged in fluid overloaded HD patients. Previous studies suggested that changes in AIx following HD session could be related to blood pressure changes and to the magnitude of fluid removal [28]. Yet, the relationship between AIx and blood pressure makes interpretation of our results challenging. Parallel changes in AIx and PWV in relation to volume changes would provide stronger support for real changes in vascular stiffness than AIx alone.

There are several physiological responses to ultrafiltration during the HD session which could obscure changes in vascular stiffness parameters. Options are activation of the sympathetic nervous system and adjustments in vascular resistance by blood flow autoregulation [29, 30]. After fluid removal, stroke volume, cardiac output, and blood pressure tend to decrease, triggering the baroreceptor reflex which leads to an increase in peripheral vascular resistance. Conversely, in response to a decrease in blood pressure, blood flow autoregulation can mediate vasodilation and a decrease in peripheral vascular resistance. Changes in vascular tension, as well and shifts in the reflection point of the pulse wave can affect measures of vascular stiffness parameters like AIx and PWV. Taken together, the combined physiological responses to the dialysis session would make it hard to predict the net effect on vascular stiffness parameters.

We hypothesized that *interdialytic* fluid accumulation could lead to an increase in vascular stiffness parameters in HD patients. Our findings could not confirm a gradual change in PWV and AIx during the 24-hours of fluid accumulation in HD patients. However, since changes in vascular stiffness parameters could be more pronounced in fluid overload versus non-fluid overloaded HD patients, we also compared these two groups. Two previous reports are available about interdialytic changes in vascular stiffness parameters in HD patients both using 48 hours measurements, yet, using two different technologies [31, 32]. These studies describe that the interdialytic PWV did not change, whereas AIx gradually increased. However, the increase in AIx was associated with increases in blood pressure [31]. Also, these two studies did not specify the fluid status of the HD patients, which limits the ability to compare results. There are several explanations for the stability of PWV and AIx during the 24-hour interdialytic period. First, since PWV is strongly related to viscoelastic remodeling of vascular wall, the 24-hour interdialytic time interval might not be sufficient to show a clear change in PWV, as suggested previously [33]. Second, a 48-hour interdialytic interval could produce more pronounced fluctuations in wave reflections, as assessed by AIx, than the 24-hour interdialytic interval [32]. Third, antihypertensive medications, which were used more frequently by fluid overloaded HD patients in our study, might obscure changes in vascular stiffness parameters [11]. From a physiological perspective, fluid overload increases blood pressure [34]. The blood pressure parameters during interdialytic period were higher in fluid overloaded HD than non-fluid overloaded HD patients. Since AIx is one of the representative measures of wave reflections, including pulse pressure, it is logical that the difference in interdialytic AIx would be more pronounced in fluid overloaded HD than non-fluid overloaded HD patients.

Although the current study provides a full description of vascular stiffness parameters changes during inter-and intradialytic fluid volume changes, there are limitations. Our small sample size could potentially be perceived as limitation; however, the study was adequately powered to demonstrate a biologically relevant change in vascular stiffness parameters. Yet, our findings are consistent with the existing literature [11, 25, 31]. We did not study vascular stiffness parameters during the 48-hour interdialytic interval which might have provided more insight in vascular stiffness parameters modulation than the 24-hour interdialytic interval. That said, to our knowledge very few studies have evaluated the effect of inter- and intradialytic fluid changes on vascular stiffness parameters and no studies have investigated this effect in fluid overloaded HD compared to non-fluid overloaded HD patients. Absence of echo cardio graphic data has slightly limited our conclusion whether cardiac issues were predominant in fluid overloaded versus non-fluid overloaded HD patients.

Another possibility is that fluid overload affects vascular function via mechanisms other than classical hemodynamics, such as by a direct influence of salt on vascular function. The endothelial glycocalyx can become functionally and structurally impaired by high sodium [35]. In addition, non-osmotic sodium storage in the skin could affect vascular function via VEGF-C. Effects of sodium on vascular function via disturbances in the glycocalyx and via dysregulation of the skin-sodium pathway are promising targets for follow up studies.

In conclusion, our study was unable to demonstrate that interdialytic fluid accumulation and intradialytic fluid removal affects vascular stiffness parameters in HD patients. The post-HD AIx reduction in non-fluid overloaded HD patients suggests that an adequate ultrafiltration volume could improve the wave reflections but not PWV. These and previous observations call for studies on chronic adjustments in fluid volume status and vascular stiffness parameters, including studies directed towards the direct effects of salt on vascular function.

## Supporting information

S1 Table Characteristics of FO and vascular stiffness parameters of the healthy individuals based on age and BMI. P value < 0.05 is considered significant; FO, fluid overload; PWV, pulse wave velocity; AIx, augmentation index.
(DOCX)

S2 Table Univariate /multivariate analysis of baseline PWV in healthy individuals. P value < 0.05 is considered significant; ESRD, end stage renal disease; FO, fluid overload; ECFV, extracellular fluid volume; ICFV, intracellular fluid volume; TBFV, total body fluid volume; PWV, pulse wave velocity; AIx, augmentation index.
(DOCX)

S3 Table Predictors of baseline PWV in HD patients using univariate and multivariate linear regression analysis. P value < 0.05 is considered significant; IDWG, intradialytic weight gain; FO, fluid overload; ECFV, extracellular fluid volume; ICFV, intracellular fluid volume; TBFV, total body fluid volume.
(DOCX)

S4 Table Hemodynamic data of pre-and post-HD session in HD patients. P value < 0.05 is considered significant; FO, Fluid overload; PWV, pulse wave velocity; AIx, augmentation index.
(DOCX)

S5 Table The correlation of net ultrafiltration (L) and intradialytic changes in vascular stiffness parameters and blood pressure measurements in HD patients. P value < 0.05 is considered significant; FO, Fluid overload; PWV, pulse wave velocity; AIx, augmentation index; net ultrafiltration, is the pre- and post- HD weight difference; delta calculated as the difference between the post- and pre-HD measurement.
(DOCX)

S1 Dataset (XLSX)
