# Correction: Inter- and intradialytic fluid volume changes and vascular stiffness parameters in patients on hemodialysis

**DOI:** 10.1371/journal.pone.0319275

**Published:** 2025-02-07

**Authors:** Aya Lafta, Judy Ukrainetz, Sara Davison, Stephanie Thompson, Aminu Bello, Branko Braam

In Table 1, the values in the section “Underlying causes of ESRD N, %” are missing. Please see the correct Table 1 here.

**Table 1:** Demographic and clinical characteristics in healthy controls and HD patients.

Parameters	Healthy controls (n = 26)	HD group (n = 39)	P value	FO HD (n = 20)	non-FO HD (n = 19)	P value
Sex M N, %	10 (38.4)	24 (61.5)	0.06	14 (70)	10 (52.6)	0.26
Age, year	49 (29–56)	60 (49–66)	0.006	62 (51–68)	57 (21–67)	0.05
Body mass index, kg/m^2^	23.9 ± 3.5	27.0 ± 5.6	0.008	26.2 ± 4.9	27.9 ± 6.2	0.33
Weight, kg	68.2 ± 10.9	78.9 ± 17.4	0.003	77.9 ± 16.9	79.9 ± 18.4	0.72
Height, cm	168.4 ± 6.8	170.0 ± 10.7	0.50	171.2 ± 10.5	168.8 ± 11.2	0.44
Post-HD weight, kg	-	77.0 ± 17.7	-	75.8 ± 16.9	78.2 ± 18.0	0.66
Underlying cause of ESRD N, %						
Diabetes	-	7 (17.9)	-	4 (20)	3 (15.7)	0.99
Hypertension	-	4 (10.2)	-	2 (10)	2 (10.5)	1.00
Polycystic kidney disease	-	2 (5.1)	-	1 (5)	1 (5.2)	1.00
Glomerulonephritis	-	2 (5.1)	-	-	2 (10.5)	0.23
Unknown	-	3 (7.6)	-	2 (10)	1 (5.2)	0.48
Other	-	21 (53.8)	-	11 (55)	10 (52.6)	0.99
FO, L	-0.2 ± 0.6	2.0 ± 2.4	<0.0001	3.7 ± 2.3	0.1 ± 0.5	< 0.0001
FO post-HD, L	-	0.0 ± 2.2	-	1.6 ± 1.9	-1.6 ± 1.0	<0.0001
ECFV, L	15.10 ± 2.1	17.88 ± 4.2	0.001	19.17± 4.2	16.54 ± 3.9	0.05
ICFV, L	19.08 ± 19.0	18.30 ± 18.3	0.482	17.55 ± 4.0	19.09 ± 4.7	0.28
TBFV, L	34.18 ± 5.9	36.10 ± 8.1	0.287	36.72 ± 7.9	35.63 ± 8.5	0.69
FO/ECFV, %	-0.7 (-4.5–3.1)	7.1 (1.6–19.5)	<0.0001	18.8 (10.0–24.9)	1.6 (-2.0–3.7)	<0.0001
IDWG, kg	-	1.3 (0.0–3.4)	-	1.4 (0.87–2.0)	1.2 (0.6–2.1)	0.66
Dialysis vintage, years	-	2.0 (0.1–12.0)	-	3.0 (2.0–4.0)	1.0 (0.3–3.0)	0.02
Net ultrafiltration, L	-	1.9 ± 1.1	-	2.2 ± 1.1	1.7 ± 1.0	0.19
Anuric N, %	-	13 (33)	-	4 (20)	9 (47)	0.07
Diabetes N, %	-	10 (2.5)	-	6 (30)	4 (21)	0.52
Cardiovascular diseases N, %	-	21 (53)	-	10 (50)	11(57)	0.61
Anti-hypertensive medications N, %	-	23 (59)	-	15 (75)	8 (42)	0.03
Beta blockers N, %	-	18 (46)	-	14 (70)	4 (21)	0.25
Calcium channel blockers N, %	-	11 (28)	-	9 (45)	3 (15.7)	0.16
Diuretics N, %	-	7 (18)	-	5 (25)	2 (10.5)	0.21
Angiotensin receptor blockers N, %	-	5 (12.8)	-	4 (20)	1 (5.2)	0.40
Baseline systolic blood pressure, mmHg	117.0 ± 11.9	138.2 ± 22.2	<0.0001	144.4 ± 21.5	131.7 ± 21.5	0.07
Baseline diastolic blood pressure, mmHg	69.3 ± 11.3	76.1 ± 15.5	0.04	76.8 ± 11.0	75.4 ± 19.5	0.78
Baseline mean arterial pressure, mmHg	85.2 ± 10.8	95.7 ± 15.4	0.002	99.4 ± 13.0	91.7 ± 17.2	0.12
Baseline heart rate, bpm	69.6 ± 12.1	79.7 ± 12.2	0.001	78.7 ± 12.2	80.8 ± 12.6	0.70
Baseline pulse pressure, mmHg	49.9 ± 9.9	61 ± 15.8	<0.001	60.0 ± 11.5	50.9 ± 15.3	0.04
Baseline PWV, m/s	8.8 ± 1.4	10.3 ± 1.7	<0.001	10.3 ± 1.4	10.2 ± 1.9	0.83
Baseline AIx, %	-22.3 ± 27.1	-10.3 ± 36.6	0.13	-14.8 ± 30.7	-5.5 ± 42.2	0.44
24hr-mean Systolic blood pressure, mmHg	-	131.6 ± 19.5	-	138.2 ± 18.0	123.5 ± 17.2	0.01
24hr-mean Diastolic blood pressure, mmHg	-	70.8 ± 11.5	-	72.1 ± 8.8	68.8 ± 13.4	0.37
24hr-mean arterial pressure, mmHg	-	90.4 ± 12.9	-	99.4 ± 13.0	91.7 ± 17.2	0.01
24hr-mean heart rate, bpm	-	78.5 ± 8.6	-	77.8 ± 10.4	79.2 ± 6.3	0.66
24hr-mean Pulse pressure, mmHg	-	60.4 ± 13.4	-	65.8 ± 14.4	54.7 ± 9.8	0.008
24hr-mean PWV, m/s	-	9.9 ± 1.1	-	9.9 ± 1.1	9.8 ± 1.3	0.80
24hr-mean AIx, %	-	-12.4 ± 22.3	-	-1.4 ± 21.9	-25.6 ± 18.1	<0.001
