# Supplementary material for: Inter- and intradialytic fluid volume changes and vascular stiffness parameters in patients on hemodialysis
Source: PLoS One. 2022 Feb 3;17(2):e0262519. doi: 10.1371/journal.pone.0262519 (PMC8812974; doi:10.1371/journal.pone.0262519)
Supplement: S1 Table — P value < 0.05 is considered significant; FO, fluid overload; PWV, pulse wave velocity; AIx, augmentation index. (DOCX) [file pone.0262519.s001.docx]

**S1 Table. Characteristics of FO and vascular stiffness parameters of the healthy individuals based on Age and BMI**

| Parameters |  |  | P value |
| --- | --- | --- | --- |
| **Age** | <49 (n=13) | >49 (n=13) |  |
| FO, L | -0.36 ± 0.7 | 0.03 ± 0.5 | 0.18 |
| Baseline PWV, m/s | 8.1 ± 0.9 | 9.5 ± 1.5 | 0.01 |
| Baseline AIx, % | -36.9 ± 23.5 | -7.8 ± 22.8 | 0.01 |
| **BMI** | <23.9  (n=16) | >23.9  (n=10) |  |
| FO, L | -0.36 ± 0.7 | 0.03 ± 0.5 | 0.11 |
| Baseline PWV, m/s | 8.9 ± 1.4 | 8.6 ± 1.3 | 0.37 |
| Baseline AIx, % | -27.1 ± 27.1 | -14.7 ± 26.6 | 0.27 |

P value < 0.05 is considered significant; FO, fluid overload; PWV, pulse wave velocity; AIx, augmentation index
