# Supplementary material for: Inter- and intradialytic fluid volume changes and vascular stiffness parameters in patients on hemodialysis
Source: PLoS One. 2022 Feb 3;17(2):e0262519. doi: 10.1371/journal.pone.0262519 (PMC8812974; doi:10.1371/journal.pone.0262519)
Supplement: S2 Table — P value < 0.05 is considered significant; ESRD, end stage renal disease; FO, fluid overload; ECFV, extracellular fluid volume; ICFV, intracellular fluid volume; TBFV, total body fluid volume; PWV, pulse wave velocity; AIx, augmentation index. (DOCX) [file pone.0262519.s002.docx]

**S2 Table. Univariate /multivariate analysis of baseline PWV in healthy individuals**

| Parameters |  |  | Univariate analysis |  |
| --- | --- | --- | --- | --- |
|  | B | T | CI (95%) | P value |
| Age, year | 0.049 | 2.94 | 4.88 - 8.21 | 0.007 |
| Body mass index, kg/m^2^ | -0.074 | -0.93 | -0.237- 0.089 | 0.35 |
| FO, L | 0.155 | 0.37 | -0.699 - 1.00 | 0.71 |
| ECFV, L | -0.265 | -2.29 | -0.503 - (-0.027) | 0.03 |
| ICFV, L | -0.149 | -2.41 | -0.278 -0.021 | 0.02 |
| TBFV, L | 0.069 | 2.09 | -0.188 - (-0.017) | 0.02 |
| ECFV/TBFV, % | 16.331 | 1.59 | -14.9 - 37.5 | 0.12 |
| ICFV/ECFV, % | 5.221 | 1.71 | -1.06 - 11.50 | 0.09 |
| Systolic blood pressure, mmHg | 0.011 | 0.44 | -0.039 - 0.061 | 0.66 |
| Diastolic blood pressure, mmHg | 0.007 | 0.26 | -0.046 - 0.059 | 0.79 |
| Mean arterial pressure, mmHg | 0.009 | 0.34 | -0.046 - 0.064 | 0.73 |
| Pulse pressure, mmHg | 0.014 | 0.49 | -0.045 -0.074 | 0.62 |
|  | Multivariate Analysis | | | |
| Age | 0.065 | 3.18 | 0.022 - 0.108 | 0.005 |

P value < 0.05 is considered significant; ESRD, end stage renal disease; FO, fluid overload; ECFV, extracellular fluid volume; ICFV, intracellular fluid volume; TBFV, total body fluid volume; PWV, pulse wave velocity; AIx, augmentation index
