# Supplementary material for: Inter- and intradialytic fluid volume changes and vascular stiffness parameters in patients on hemodialysis
Source: PLoS One. 2022 Feb 3;17(2):e0262519. doi: 10.1371/journal.pone.0262519 (PMC8812974; doi:10.1371/journal.pone.0262519)
Supplement: S3 Table — P value < 0.05 is considered significant; IDWG, intradialytic weight gain; FO, fluid overload; ECFV, extracellular fluid volume; ICFV, intracellular fluid volume; TBFV, total body fluid volume. (DOCX) [file pone.0262519.s003.docx]

**S3 Table. Predictors of baseline PWV in HD patients using univariate and multivariate linear regression analysis**

| Parameters |  |  | Univariate analysis |  |
| --- | --- | --- | --- | --- |
|  | B | T | CI (95%) | P value |
| Age, year | 0.037 | 1.939 | -0.002-0.076 | 0.06 |
| Body mass index, kg/m^2^ | -0.011 | -0.218 | -0.112-0.090 | 0.82 |
| Pre-HD weight, kg | 0.026 | 1.691 | -0.005-0.058 | 0.09 |
| IDWG (kg) | -0.22 | -0.696 | -0.886-0.433 | 0.49 |
| FO, L | 0.088 | 0.780 | -0.141-0.317 | 0.45 |
| ECFV, L | 0.132 | 2.113 | 0.005-0.259 | 0.04 |
| FO / ECFV, % | 1.387 | 0.573 | -3.515-6.290 | 0.57 |
| ICFV, L | 0.111 | 1.817 | -0.013-0.234 | 0.07 |
| TBFV, L | 0.069 | 2.098 | 0.002-0.135 | 0.04 |
| ECFV/TBFV, % | 2.661 | 0.389 | -11.18-16.51 | 0.69 |
| ICFV/ECFV, % | 0.233 | 0.137 | -3.22-3.69 | 0.89 |
| Systolic blood pressure, mmHg | 0.015 | 1.24 | -0.01-0.041 | 0.22 |
| Mean arterial pressure, mmHg | 0.022 | 1.21 | -0.014-0.058 | 0.23 |
| Pulse pressure, mmHg | 0.027 | 1.146 | -0.021-0.075 | 0.25 |
| Diabetic N, % | 1.174 | 1.930 | -0.05-2.40 | 0.06 |
| Anti-hypertensive medications N, % | 0.540 | 0.965 | -0.59-1.67 | 0.34 |
| Dialysis vintage, years | -0.039 | -0.373 | -0.25-0.172 | 0.71 |
| Net ultrafiltration, L | 0.167 | 0.626 | -0.373-0.706 | 0.53 |
|  | Multivariate Analysis | | | |
| ECFV, L | 4.006 | 1.022 | -3.954-11.957 | 0.74 |
| TBFV, L | -3.945 | -0.997 | 0.053-3.04 | 0.38 |

P value < 0.05 is considered significant; IDWG, intradialytic weight gain; FO, fluid overload; ECFV, extracellular fluid volume; ICFV, intracellular fluid volume; TBFV, total body fluid volume.
