# Supplementary material for: Inter- and intradialytic fluid volume changes and vascular stiffness parameters in patients on hemodialysis
Source: PLoS One. 2022 Feb 3;17(2):e0262519. doi: 10.1371/journal.pone.0262519 (PMC8812974; doi:10.1371/journal.pone.0262519)
Supplement: S4 Table — P value < 0.05 is considered significant; FO, Fluid overload; PWV, pulse wave velocity; AIx, augmentation index. (DOCX) [file pone.0262519.s004.docx]

**S4 Table. Hemodynamic data of pre-and post-HD session in HD patients**

| Parameters | FO HD (n=20) | | | non-FO HD (n=19) | | |
| --- | --- | --- | --- | --- | --- | --- |
|  | Pre-HD | Post-HD | P value | Pre-HD | Post-HD | P value |
| PWV, m/s | 10.3 ± 1.4 | 9.6 ± 2.5 | 0.99 | 10.2 ± 1.9 | 10.4 ± 2.0 | 0.99 |
| AIx, % | -14.8 ± 30.7 | -20.1 ±5 3.1 | 0.89 | -5.5 ± -42.2 | -16.3 ± 39.5 | 0.04 |
| Systolic blood pressure, mmHg | 144.4 ± 21.5 | 143.1 ± 33.9 | 0.99 | 131.7 ± 21.5 | 131.4 ± 24.3 | 0.99 |
| Diastolic blood pressure, mmHg | 76.8 ± 11.0 | 75.8 ± 17.3 | 0.99 | 75.4 ± 19.5 | 76.0 ± 19.2 | 0.99 |
| Mean arterial pressure, mmHg | 99.4 ± 13.0 | 97.3 ± 21.1 | 0.99 | 91.7 ± 17.1 | 93.7 ± 19.8 | 0.99 |
| Pulse pressure, mmHg | 66.7 ± 17.7 | 64.4 ± 23.5 | 0.97 | 56.3 ± 11.9 | 53.7 ± 11.4 | 0.41 |
| Heart rate, b/m | 78.7 ±12.2 | 77.4 ± 11.9 | 0.86 | 80.8 ± 12.6 | 82.5 ± 14.1 | 0.99 |

P value < 0.05 is considered significant; FO, Fluid overload; PWV, pulse wave velocity; AIx, augmentation index
