# Supplementary material for: Inter- and intradialytic fluid volume changes and vascular stiffness parameters in patients on hemodialysis
Source: PLoS One. 2022 Feb 3;17(2):e0262519. doi: 10.1371/journal.pone.0262519 (PMC8812974; doi:10.1371/journal.pone.0262519)
Supplement: S5 Table — P value < 0.05 is considered significant; FO, Fluid overload; PWV, pulse wave velocity; AIx, augmentation index; net ultrafiltration, is the pre- and post- HD weight difference; delta calculated as the difference between the post- and pre-HD measurement. (DOCX) [file pone.0262519.s005.docx]

**S5 Table. The correlation of net ultrafiltration (L) and intradialytic changes in vascular stiffness parameters and blood pressure measurements in HD patients**

| Parameters | HD group (n=39) | | FO HD group (n=20) | | non-FO HD group (n=19) | |
| --- | --- | --- | --- | --- | --- | --- |
|  | r^2^ | P value | r^2^ | P value | r^2^ | P value |
| Post-HD PWV, m/s | 0.033 | 0.26 | 0.033 | 0.44 | 0.082 | 0.23 |
| delta PWV, m/s | 0.108 | 0.04 | 0.046 | 0.35 | 0.300 | 0.01 |
| Post-HD AIx, % | 0.019 | 0.40 | 0.193 | 0.05 | 0.109 | 0.16 |
| delta AIx, % | 0.002 | 0.75 | 0.088 | 0.20 | 0.082 | 0.23 |
| Post-HD Systolic blood pressure, mmHg | 0.042 | 0.20 | 0.093 | 0.19 | 0.003 | 0.81 |
| Post-HD Diastolic blood pressure, mmHg | 0.0008 | 0.86 | <0.001 | 0.99 | 0.003 | 0.80 |
| Post-HD Mean arterial pressure, mmHg | 0.005 | 0.66 | 0.015 | 0.60 | 0.001 | 0.88 |
| Post-HD Pulse pressure, mmHg | 0.157 | 0.01 | 0.235 | 0.02 | 0.006 | 0.75 |

P value < 0.05 is considered significant; FO, Fluid overload; PWV, pulse wave velocity; AIx, augmentation index; net ultrafiltration, is the pre- and post- HD weight difference; delta calculated as the difference between the post- and pre-HD measurement
